# Targeted Intracellular Delivery of Amino Acids to Trophoblast Cells Reveals Proteomic Signatures of Cellular Utilisation

**DOI:** 10.3390/biom16050628

**Published:** 2026-04-23

**Authors:** Emily Mazey, Sarah Flannery, Roman Fischer, Neva Kandzija, Wei Zhang, Yuma Yamada, Manabu Tokeshi, Errin Johnson, Naveed Akbar, James Bancroft, Fadil M. Hannan, Manu Vatish

**Affiliations:** 1 Nuffield Department of Women’s and Reproductive Health, University of Oxford, Oxford OX3 9DU, UKmanu.vatish@wrh.ox.ac.uk (M.V.); 2 Target Discovery Institute, Nuffield Department of Medicine, University of Oxford, Oxford OX3 7FZ, UK; 3 Faculty of Pharmaceutical Sciences, Hokkaido University, Sapporo 060-0812, Japan; 4 Faculty of Engineering, Hokkaido University, Sapporo 060-0813, Japan; 5 Electron Microscopy Facility, Sir William Dunn School of Pathology, University of Oxford, Oxford OX1 3RE, UK; 6 Division of Cardiovascular Medicine, Radcliffe Department of Medicine, University of Oxford, Oxford OX3 9DU, UK; 7 Cellular Imaging Core Facility, Centre for Human Genetics, Nuffield Department of Medicine, University of Oxford, Oxford OX3 7BN, UK

**Keywords:** placenta-targeted delivery, liposomes, placental chondroitin sulfate-A-binding peptide (plCSA-bp), trophoblast cells, amino acid delivery, SILAC proteomics, quantitative proteomics

## Abstract

Targeted delivery systems offer a promising approach for selectively modulating cellular processes; yet the intracellular consequences of targeted nutrient delivery to trophoblast cells remain poorly defined. Here, we investigated a previously validated placenta-targeting peptide conjugated to liposomes encapsulating stable isotope-labelled L-arginine and L-lysine to examine cellular uptake and downstream molecular responses in a trophoblast-like cell model. Peptide-dependent uptake of fluorescently labelled liposomes was confirmed in BeWo cells, demonstrating selective internalisation compared with non-targeted controls. Encapsulation of isotope-labelled amino acids enabled direct quantification of intracellular delivery and incorporation into the cellular proteome using stable isotope labelling by amino acids in cell culture (SILAC). Quantitative proteomic analysis revealed coordinated changes in proteins associated with translation, metabolism, and nitric oxide synthase regulation following targeted liposomal uptake. Notably, V-type proton ATPase subunit G1 (ATP6V1G1) and large neutral amino acid transporter small subunit 1 (SLC7A5) showed increased incorporation of labelled amino acids and were independently validated by Western blotting. Together, these findings establish a proof-of-concept platform for targeted intracellular amino acid delivery to trophoblast-like cells and define the resulting proteomic responses. This work provides mechanistic insight into intracellular amino acid utilisation and a framework for future studies in placental cell biology.

## 1. Introduction

The placenta plays a critical role in supporting foetal development by regulating the transfer of nutrients, oxygen, and hormones between the maternal and foetal circulations [1]. Amino acids are of particular importance, serving as substrates for placental metabolism, protein synthesis, and foetal growth [2,3]. Consequently, placental amino acid processing is tightly regulated, and disruptions to these processes can have significant effects on placental and foetal development [4,5,6,7].

In pregnancy complications, including foetal growth restriction (FGR), placental nutrient availability is compromised due to both reduced placental perfusion and impaired transport-mediated uptake, limiting delivery to placental cells and the foetus [4,5,6,8,9,10,11]. This has led to growing interest in strategies that could enhance placental nutrient delivery or utilisation. However, conventional approaches such as maternal amino acid supplementation rely on intact placental transport mechanisms and have shown variable efficacy, highlighting the need for alternative delivery strategies that do not depend solely on endogenous transporter activity [12,13,14,15,16,17,18].

Nanoparticle-based delivery systems, such as liposomes, provide a versatile platform for targeted delivery of therapeutic or nutritional payloads to specific tissues [19,20,21,22,23]. Liposomes can be functionalised with targeting ligands to promote selective cellular uptake [24,25,26]. For placental targeting, the peptide EDVKDINFDTKEKFLAGCLIVSFHEGKC (EC), also known as the placental chondroitin sulphate A-binding peptide (plCSA-bp), binds chondroitin sulphate A on trophoblast cells and has demonstrated placental specificity in vivo, making it an attractive candidate for placenta-directed delivery [27,28,29,30].

While previous studies have established the placenta-targeting capabilities of the EC peptide, the intracellular consequences of targeted nutrient delivery remain largely unexplored. In particular, it is unclear how selectively delivered amino acids are incorporated into the cellular proteome and whether targeted delivery triggers coordinated molecular responses within trophoblast cells.

In this study, we investigated the previously validated EC placenta-targeting peptide conjugated to liposomes encapsulating stable isotope-labelled L-arginine and L-lysine in a trophoblast-like cell model [31,32]. Arginine and lysine were selected for their dual relevance to placental physiology and quantitative proteomic analysis. Arginine, a semi-essential amino acid, supports protein synthesis and serves as a substrate for nitric oxide, a vasodilator that promotes placental blood flow and nutrient delivery [33,34,35,36,37,38]. Lysine, an essential amino acid, contributes to protein synthesis, collagen formation, and the structural integrity of trophoblasts, supporting placental development and nutrient uptake [39,40,41]. In addition, both amino acids are ideal for stable isotope labelling by amino acids in cell culture (SILAC) experiments, as they enable precise quantification via mass spectrometry due to their susceptibility to trypsin cleavage [42,43].

SILAC is a quantitative proteomics technique that tracks the incorporation of heavy isotope-labelled amino acids into the proteome via mass spectrometry [44,45]. In conventional SILAC, cells are cultured in media containing labelled amino acids [46]. Here, we adapted this approach for direct delivery by encapsulating SILAC L-arginine (R10) and L-lysine (K8) in placenta-targeted liposomes and administering them to trophoblast-like cells. This strategy enables direct quantification of intracellular amino acid delivery and proteomic incorporation, providing a functional readout of peptide targeting efficiency.

The objectives of our study were to (i) confirm peptide-mediated cellular uptake of liposomes, (ii) quantify intracellular delivery and incorporation of amino acids using SILAC, and (iii) define downstream proteomic changes associated with targeted amino acid delivery. By focusing on intracellular uptake and molecular responses under baseline conditions, this work establishes a proof-of-concept framework for placenta-targeted amino acid delivery and provides mechanistic insight relevant to future studies of placental function.

## 2. Materials and Methods

### 2.1. DiI Liposomes and SILAC Liposomes Preparation

Liposomes were prepared using a microfluidic device (Faculty of Engineering and Faculty of Pharmaceutical Sciences, Hokkaido University) based on a previously established method and lipid formulation [47]. A lipid phase of 1,2-Dioleoyl-sn-glycero-3-phosphoethanolamine (Santa Cruz Biotechnology, Dallas, TX, USA), porcine brain sphingomyelin (Avanti Polar Lipids, Alabaster, AL, USA), and 1,2-distearoyl-sn-glycero-3-phosphoethanolamine-N-[methoxy(polyethylene glycol)-2000] (Avanti Polar Lipids) (9:2:0.33 molar ratio) was infused into an aqueous phase consisting of phosphate-buffered saline (PBS; Sigma-Aldrich, Burlington, MA, USA, D8537; 1×, 0.2 g/L KCl, 0.2 g/L KH_2_PO_4_, 8.0 g/L NaCl, 1.15 g/L Na_2_HPO_4_) at 100 µL/min and 400 µL/min, respectively. Liposomes were prepared using this defined lipid composition, and the final liposome concentration reflects the initial lipid input used during microfluidic formulation.

For DiI liposomes, 1,1′-Dioctadecyl-3,3,3′,3′-tetramethylindocarbocyanine perchlorate (DiI) (Insight Biotechnology, London, UK) was incorporated at a 0.055 molar ratio. For SILAC liposomes, the aqueous phase contained 14.2 mM L-arginine-HCl 13C6 (heavy arginine, R10) (Thermo Scientific, Waltham, MA, USA) and 28.5 mM L-lysine-2HCl 4,4,5,5-D4 (heavy lysine, K8) (Thermo Scientific) in PBS. Empty control liposomes and DiI liposomes contained PBS alone in the aqueous phase. Filtration for purification of liposomes was performed using Amicon Ultra-4 100 kDa centrifugal filters (Millipore, Burlington, MA, USA) at 2000× *g* for 10 min and resuspended in fPBS to the starting volume. Liposomes were stored at 4 °C and used within 24 h of preparation.

### 2.2. Peptide Conjugation

Lyophilised stearylated peptides, including EC (ProteoGenix S.A.S., Schiltigheim, France), scrEC (EVDNDKKLGLVFEKDKIFTEFACISHCG) (ProteoGenix S.A.S.), and R8 (RRRRRRRR) (Toray Industries, Tokyo, Japan), were reconstituted at 2 mg/mL in fPBS, mixed with liposomes at a 1:10 ratio, vortexed, and incubated for 30 min at room temperature. Unlabelled liposomes were incubated with fPBS only at the same ratio.

### 2.3. Transmission Electron Microscopy (TEM)

Imaging was performed using a JEOL 1400 TEM at 120 kV with a Gatan Rio CMOS camera (Sir William Dunn School of Pathology, University of Oxford, Oxford, UK). DiI liposomes were applied to carbon formvar 300-mesh copper grids, negatively stained with 2% uranyl acetate for 10 s, and air-dried before imaging.

### 2.4. Nanoparticle Tracking Analysis (NTA)

NTA was performed using a NanoSight NS500 instrument (Malvern Panalytical, Malvern, UK) with NTA software (v3.4). Three independent DiI liposome preparations were diluted 1:250 in fPBS, and five 1 min microscopy image videos were recorded per sample under identical acquisition settings for all measurements, as previously described [48]. Particle size distribution and concentration were analysed using the NTA software, and data were visualised using GraphPad Prism (v10).

### 2.5. Zeta Potential Measurements

Zeta potential, an indicator of surface charge and colloidal stability, was measured to evaluate the electrostatic properties of DiI liposomes and assess the impact of peptide conjugation. Surface charge influences liposome stability, interaction with biological membranes, and cellular uptake, making it a critical parameter for nanoparticle drug delivery.

Measurements were performed using a ZetaView instrument (Particle Metrix, Inning am Ammersee, Germany) (Radcliffe Department of Medicine, University of Oxford). Three independent DiI liposome preparations, with and without peptide conjugation, were diluted 1:10,000 in deionised water. Prior to analysis, calibration alignment was performed using 100 nm polymer calibration beads (Applied Microspheres, Mainz, Germany). Samples were analysed using a 488 nm laser in stationary 2-cycle mode, scanning 11 positions per cycle under identical acquisition settings for all measurements. Analysis parameters included a minimum brightness of 20, a minimum particle area of 10 pixels, and a maximum area of 1000 pixels. Zeta potential was calculated using ZetaView software (v8.05.12) based on electrophoretic mobility using the Henry/Smoluchowski equation. Statistical analysis included a Shapiro–Wilk test for normality, followed by an unpaired two-tailed *t*-test to determine statistical significance between plain and peptide-conjugated liposomes, with a *p*-value cut-off of 0.05. Data were analysed and visualised using GraphPad Prism (v10).

### 2.6. SILAC Encapsulation Efficiency

Encapsulation efficiency of SILAC amino acids in liposomes was determined by measuring amino acid concentrations in two fractions: the liposome fraction (after lysis), representing the encapsulated amino acids, and the filtrate fraction containing the free (non-encapsulated) amino acids. Measurements were performed using an L-amino acid quantitation assay (Sigma-Aldrich).

Liposomes were lysed by a 1:2 dilution with methanol (Fisher Chemical, Waltham, MA, USA), sonicated for 5 min, and then further diluted 1:12 with assay buffer (final dilution: 1:24). The filtrate, obtained after separation of liposomes by filtration as detailed in the DiI Liposome and SILAC Liposomes Preparation section of the methods, was diluted 1:44 with assay buffer. Standards (1 mM–200 µM), blank controls, and samples were prepared in a 96-well clear plate (Thermo Scientific), mixed 1:1 with assay master mix, incubated at 37 °C for 30 min, and absorbance measured at 570 nm using a CLARIOstar plate reader (BMG Labtech, Ortenburg, Germany). Data analysis was performed using CLARIOstar MARS software (v3.31) and Microsoft Excel. Encapsulation efficiency (%) was calculated as the proportion of amino acids encapsulated within liposomes relative to the total amino acids (sum of encapsulated plus free amino acids), using the formula:

Encapsulation Efficiency (%) = ((Encapsulated amino acid concentration (in lysed liposomes)))/((Encapsulated amino acid concentration + Free amino acid concentration (in filtrate))) × 100

### 2.7. BeWo Cell Culture and Liposome Treatments

All experiments were conducted in vitro using BeWo cells (ECACC; Cellosaurus; CVCL_0044), a human choriocarcinoma cell line commonly used as a model for the trophoblast layer of the placenta due to its ability to retain key features of trophoblasts, including nutrient transport [31,32]. BeWo cells were selected as a well-established trophoblast-like model that enables controlled mechanistic and proteomic investigations of intracellular processes relevant to placental function. The cells were cultured in DMEM (4.5 g/L D-glucose, 10% FBS, 2 mM glutamine, 1% penicillin-streptomycin) (Sigma-Aldrich) at 37 °C, 21% O_2_, and 5% CO_2_.

For fluorescence microscopy, cells (n = 9, comprising three independent cultures with three technical replicates per culture) were seeded at 4 × 10^4^ cells/cm^2^ in 8-well chamber slides (Lab-Tek, Waltham, MA, USA) and treated with EC-labelled (placenta-targeting), scrEC-labelled (negative control), R8-labelled (positive control; cationic peptide known to enhance non-specific cellular uptake [49]), or plain DiI liposomes, or PBS (no treatment), diluted 1:10 in DMEM, for 2 h. Following treatment, cells were washed with 40 IU/mL heparin (Wockhardt, Mumbai, India) and PBS, stained with 5 µg/mL wheat germ agglutinin Alexa-Fluor 488 (Invitrogen, Waltham, MA, USA) for 10 min, fixed with 4% paraformaldehyde (Thermo Scientific) for 10 min, and mounted with DAPI-containing mounting medium (Abcam, Cambridge, UK).

For SILAC proteomics, cells (n = 3 technical replicates) were seeded at 3.5 × 10^4^ cells/cm^2^ in 60 mm dishes (Sarstedt, Numbrecht, Germany) and treated with EC-labelled SILAC liposomes or plain SILAC liposomes, diluted 1:10 in SILAC DMEM (Thermo Scientific) supplemented with 10% dialysed FBS (Gemini Biosciences, Liverpool, UK) and 1% penicillin/streptomycin (Sigma-Aldrich) (final SILAC amino acid concentration: 1.2 mM). A positive control group received empty liposomes diluted 1:10 in SILAC DMEM supplemented with 440 µM R10 and 890 µM K8 SILAC amino acids. Cells were incubated with treatment conditions for 6 h, then washed with 40 IU/mL heparin and PBS, and incubated for an additional 18 h in SILAC DMEM without liposomes or SILAC amino acids prior to protein extraction and mass spectrometry analysis.

### 2.8. Fluorescent Microscopy

BeWo cells were imaged using an SP8 FALCON confocal microscope (Leica Microsystems, Wetzlar, Germany,) equipped with LAS-X software (Leica Microsystems, v5.1) (Centre for Human Genetics, University of Oxford). Images were acquired using a 60× water immersion objective (1.3 NA), 1024 × 1024 pixel scan size, 600 Hz scan speed, and Leica prism-based spectral dispersion for spectral filtering. Fluorophores were excited using the following laser lines and detection systems: DAPI was imaged using a 405 nm (violet) laser and detected with a HyD detector; wheat germ agglutinin Alexa-Fluor 488 was excited with a 488 nm (blue) laser and detected with a PMT detector; and DiI was excited using a 561 nm (yellow-green) laser and detected with a HyD detector. All acquisitions were performed under identical settings.

### 2.9. Protein Extraction and Mass Spectrometry

Cells were washed with ice-cold fPBS, lysed in ice-cold RIPA buffer (Thermo Scientific) with protease inhibitor (Roche, Basel, Switzerland) and Pierce phosphatase inhibitor (Thermo Scientific) for 20 min, agitated with a 23 G needle, and centrifuged at 15,000× *g* at 4 °C for 20 min. Protein concentration was determined using a Pierce BCA assay (Thermo Scientific).

Mass spectrometry was performed by the Discovery Proteomics Group (Target Discovery Institute, University of Oxford). Protein samples were denatured in 8 M urea in 100 mM tetraethylammonium bromide and processed using filter-aided sample preparation. Proteins were digested overnight at 37 °C with trypsin (Promega, Madison, WI, USA) at a 1:25 enzyme-to-protein ratio. Peptides were dried by vacuum centrifugation and reconstituted in 3% acetonitrile and 0.1% formic acid prior to analysis.

Peptides were separated by liquid chromatography using a Dionex UltiMate 3000 UHPLC system (Thermo Fisher Scientific). Samples were initially loaded onto a PepMap C18 trap column (Thermo Fisher Scientific) at a flow rate of 20 µL/min for 1 min and then separated on a 20 cm EasySpray analytical column (Thermo Fisher Scientific). Peptides were eluted over a 60 min linear gradient from 2–35% acetonitrile in 0.1% formic acid with 5% dimethyl sulfoxide at a flow rate of 250 nL/min.

Mass spectrometry was performed on a Q Exactive Orbitrap mass spectrometer (Thermo Fisher Scientific) operating in data-dependent acquisition mode. Full MS scans were acquired in the Orbitrap over a mass-to-charge ratio range of 380–1500 at a resolution of 70,000 with an automatic gain control (AGC) target of 3 × 10^6^ ions. The top 15 most intense precursor ions with a charge state of ≥2 were selected for fragmentation using higher-energy collisional dissociation with a normalised collision energy of 28. MS/MS spectra were acquired in the Orbitrap at a resolution of 17,500 with an AGC target of 1 × 10^5^ ions, a maximum acquisition time of 128 milliseconds, and a dynamic exclusion time of 27 s.

### 2.10. Proteomics Data Analysis

Data were processed with MaxQuant (Max-Planck Institute of Biochemistry) using the UniProt human proteome database (UP000005640). Protein identification was supported by unique peptide assignments as determined using MaxQuant, with all proteins exhibiting statistically significant changes identified by multiple unique peptides. Quantification was performed using isotopic labelling with a maximum of three R10/K8 amino acids per peptide and a minimum ratio count of two.

The heavy-to-light (H/L) ratio represents the relative incorporation of isotope-labelled (“heavy”) amino acids compared to the natural (“light”) amino acids in proteins, providing a quantitative measure of amino acid delivery and incorporation. Higher H/L ratios indicate greater amino acid uptake and incorporation into the proteome, providing a quantitative measure of delivery by the liposomes. For each condition, the average percentage of heavy amino acids incorporated into proteins was calculated using the following formula:

Percentage of heavy amino acid incorporation = (Heavy amino acid intensity/(Heavy amino acid intensity + Light amino acid intensity)) × 100.

Perseus software (Max-Planck Institute of Biochemistry, v2.0.10) was used for analysis, with log2-transformation of heavy-to-light ratios and imputation of missing values from a downshifted normal distribution (width 0.8, downshift 1.8). Data distribution was assessed using histograms and principal component analysis (Figures S1 and S2). Significant proteins were identified via one-way ANOVA and visualised using volcano plot and heat map (Z-score transformation and hierarchical clustering) analyses (Figures S3 and S4). A post-hoc Tukey’s test determined statistical significance between EC-labelled and plain SILAC liposome conditions.

The number of unique peptides per protein and representative peptide sequences for proteins identified as significantly increased in H/L ratio in the EC-labelled SILAC liposome condition (post-hoc Tukey’s test) are provided in the Supplementary Material (Table S1).

### 2.11. Western Blot Validation

Western blotting was performed using Jess Simple Western (Bio-Techne, Minneapolis, MN, USA). ATP6V1G1 and SLC7A5 were detected in BeWo cell lysates using primary antibodies (ATP6V1G1: 1:4 dilution, Santa Cruz Biotechnology; SLC7A5: 1:100 dilution, Cell Signalling Technology, Danvers, MA, USA), followed by HRP-conjugated secondary antibodies and luminol-peroxidase detection. Positive controls were HeLa and A549 cell lysates for ATP6V1G1 and SLC7A5, respectively.

Protein quantification was normalised to total protein using the Jess RePlex reagent and total protein detection module and analysed with Compass for Simple Western software (Bio-Techne, v6.1). The percentage difference between EC-labelled and plain SILAC liposome conditions was calculated. Statistical analysis included a Shapiro–Wilk test for normality, followed by an unpaired two-tailed *t*-test to determine statistical significance, with a *p*-value cut-off of 0.05. Data were analysed and visualised using GraphPad Prism (v10).

### 2.12. Functional Enrichment Analysis

Protein interaction network analysis was performed using STRING (Search Tool for the Retrieval of Interacting Genes/Proteins) on 22 proteins identified in the post-hoc Tukey’s test as having a significant increase in H/L ratio in the EC-labelled SILAC liposomes condition compared to the plain SILAC liposomes condition [50]. Although 23 proteins were initially identified, one protein (ANXA2P2), a pseudogene of ANXA2, was excluded because it does not encode a functional protein recognised by STRING. This analysis was conducted for *Homo sapiens*, assessing enriched molecular functions, as well as biological processes, subcellular localisations, and co-expression analyses (Figures S5–S8).

## 3. Results

### 3.1. DiI Liposome Characterisation

**Figure 1:**
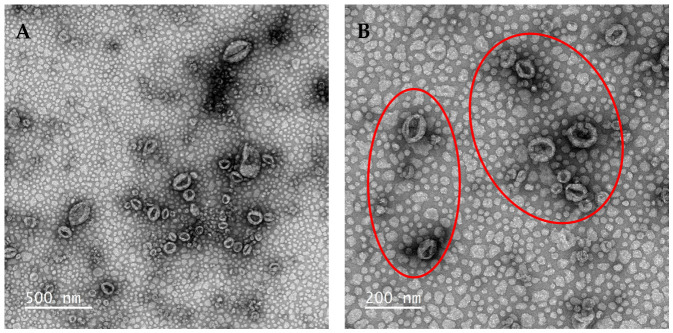
Transmission electron microscopy (TEM) images of DiI liposomes. DiI liposomes were negatively stained with uranyl acetate and imaged using a JEOL 1400 TEM at 120 kV with a Gatan Rio CMOS camera. (**A**) Scale bar = 500 nm, (**B**) scale bar = 200 nm. Red rings highlight the liposomes.

**Figure 2:**
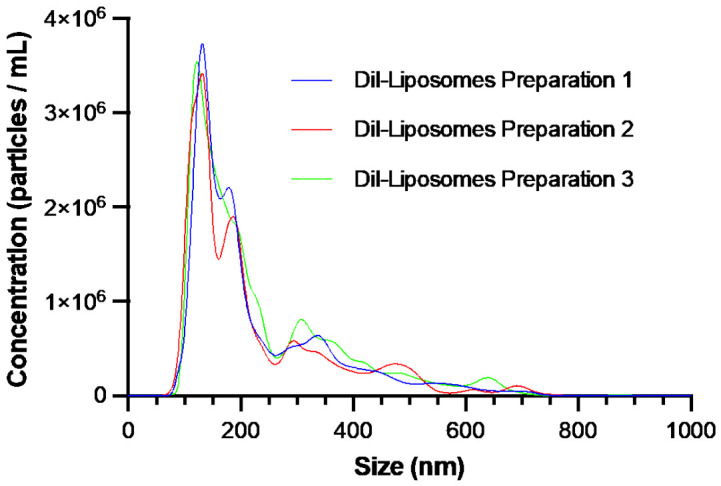
Size distribution of DiI liposomes. The size distribution of three preparations of DiI liposomes was measured at a 1:250 dilution. Analysis was conducted using NanoSight NS500.

DiI liposomes were characterised for morphology, size, and surface charge to assess their suitability for placental targeting (Figure 1 and Figure 2, Table 1).

Transmission electron microscopy (TEM) revealed predominantly ovoid and roughly spherical structures, consistent with previous reports of conventional TEM imaging of liposomes [51,52]. The observed particle size was approximately 100 nm.

Nanoparticle tracking analysis (NTA) showed a mean modal size of 128.2 ± 5.49 nm, with moderate heterogeneity observed across the liposome population. This size range was within the target threshold of <150 nm, selected to balance particle stability with efficient cellular uptake [53].

Zeta potential measurements demonstrated that plain DiI liposomes carried a negative surface charge (−22.42 ± 1.97 mV). Peptide conjugation significantly altered the surface charge: EC-labelling resulted in a more negative zeta potential (−31.32 ± 2.72 mV; *p* 0.01), as did scrambled EC (scrEC)-labelling (−30.42 ± 1.49 mV; *p* 0.0049), whereas octaarginine (R8)-labelling positively shifted the charge (+22.05 ± 2.10 mV; *p* < 0.0001). While a positive surface charge encourages non-specific cellular uptake, a negative surface charge was desired for placental delivery to minimise non-specific cellular interactions and favour selective receptor-mediated uptake by placental cells [54,55].

### 3.2. Trophoblast Cell Targeting of EC-Labelled DiI Liposomes

**Figure 3:**
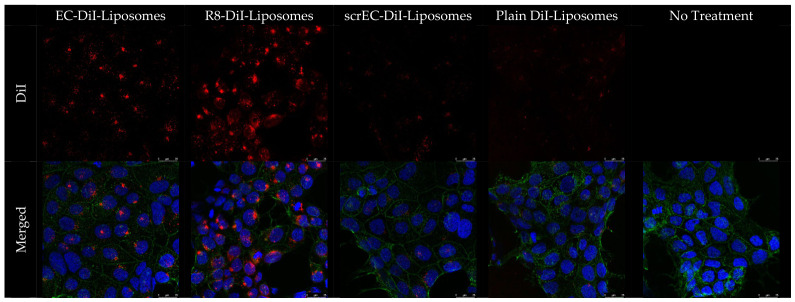
Uptake of DiI-labelled liposomes in BeWo cells (63× magnification). BeWo cells were incubated for 2 h with DiI liposomes labelled with EC peptide (placenta-targeting), R8 peptide (positive control), or scrEC peptide (negative control), plain DiI liposomes, or no treatment. Images were captured using a Leica SP8 FALCON point scanning fluorescence microscope at 60× magnification. Representative images are shown. Scale bar: 25 µm. Top row: DiI-labelled liposomes (red). Bottom row: merged images with DiI-labelled liposomes (red), DAPI-stained nuclei (blue), and wheat germ agglutinin Alexa-fluor 488-labelled cell membranes (green).

The uptake of EC-labelled DiI liposomes was validated in BeWo trophoblast-like cells using fluorescence microscopy to assess peptide-mediated targeting and cellular internalisation in this placental cell model (Figure 3). Cells treated with EC-labelled DiI liposomes exhibited robust intracellular red fluorescence, with a punctate, vesicular distribution, consistent with endocytic uptake. R8-labelled liposomes, used as a positive control, also showed high levels of intracellular fluorescence. In contrast, cells treated with scrEC-labelled or plain DiI liposomes, used as negative controls, displayed minimal fluorescence, comparable to untreated control cells. These findings demonstrate selective uptake of EC-labelled liposomes in this trophoblast cell model and support their suitability for placenta-targeted delivery in the experimental conditions used.

### 3.3. Encapsulation Efficiency of SILAC Amino Acids

Encapsulation efficiency of SILAC-labelled amino acids was assessed by quantifying amino acid concentrations in the liposome fraction (encapsulated amino acids) and the corresponding filtrate (free, non-encapsulated amino acids). SILAC liposomes achieved an encapsulation efficiency of 36%, with 11.7 mM amino acids encapsulated relative to 33.0 mM remaining free in the filtrate (Table 2). This level of encapsulation was sufficient to support downstream delivery and proteomic analyses.

### 3.4. Proteomics Consequences of Targeted SILAC Amino Acid Delivery

Following treatment with SILAC liposomes, BeWo cell lysates were analysed by quantitative proteomics to assess delivery and incorporation of isotope-labelled amino acids into the cellular proteome. Heavy-to-light (H/L) amino acid ratios were calculated to assess incorporation of isotope-labelled (“heavy”) arginine and lysine compared to endogenous (“light”) amino acids into cellular proteins. In this context, higher H/L ratios indicate greater incorporation of delivered heavy amino acids into the cellular proteome, providing a quantitative measure of delivery by the liposomes. Across all conditions, approximately 8–10% of amino acids incorporated into the proteome were heavy-labelled. EC-labelled SILAC liposome-treated cells showed an average incorporation of 8.6–8.7%, compared to 8.2–8.3% in plain SILAC liposome-treated cells and 9.2–9.6% in SILAC controls.

To identify proteins exhibiting differential amino acid incorporation across conditions, one-way ANOVA was performed on log2-tranformed H/L ratios. Differential incorporation (log2 fold-change differences) between EC-labelled and plain SILAC liposome conditions was visualised using volcano plot analysis (Table 3, Figure S3). Nine proteins showed modest but statistically significant increases in heavy amino acid incorporation in the EC-labelled SILAC liposome condition, including V-type proton ATPase subunit G1(ATP6V1G1; log2 fold-change 1.489; ≈2.8-fold increase; −log *p* 3.658) and large neutral amino acid transporter small subunit 1 (SLC7A5; log2 fold-change 0.207; ≈1.15-fold increase; −log *p* 2.066). Conversely, four proteins exhibited reduced incorporation in the EC-labelled SILAC liposome condition, including basal cell adhesion molecule (BCAM; log2 fold-change −1.574; ≈0.33-fold decrease; −log *p* 3.979) and 60S ribosomal protein L18a (RPL18; log2 fold-change −0.382; ≈0.76-fold decrease; −log *p* 1.769).

Patterns of protein incorporation across conditions are also shown in the heat map presented in the Supplementary Information (Figure S4).

Post-hoc Tukey’s testing identified 23 proteins with modest but statistically significant increases in heavy amino acid incorporation in the EC-labelled SILAC liposomes condition compared with the plain SILAC liposomes condition (Table 4). These proteins included factors involved in amino acid transport, protein folding, translation, and RNA binding, indicating a selective and coordinated proteomic response to targeted amino acid delivery. The number of unique peptides per protein and representative peptide sequences for these statistically significant proteins are provided (Table S1), demonstrating peptide-level support for protein identification.

### 3.5. Western Blot Validation

**Figure 4:**
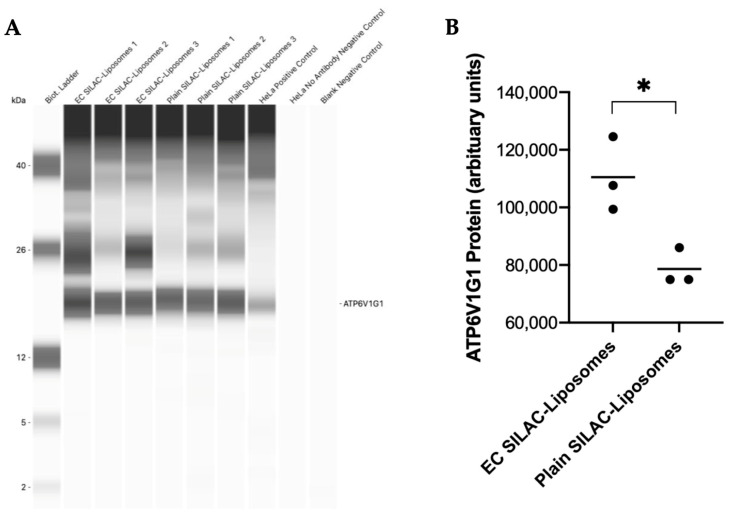
Western blot analysis and quantification of ATP6V1G1 protein expression. Western blot analysis of ATP6V1G1 in BeWo cell lysates following treatment with EC-labelled SILAC liposomes or plain SILAC liposomes, performed using Jess Simple Western (**A**). HeLa cell lysate served as a positive control, while HeLa cell lysate without ATP6V1G1 antibody incubation and a no-sample control served as negative controls. A 1 mg/mL protein concentration and a 1:4 ATP6V1G1 antibody dilution were used. A biotinylated molecular weight ladder confirmed a 19 kDa ATP6V1G1 band. Quantification of ATP6V1G1, normalised to total protein, was performed using Compass for Simple Western software, with results displayed in arbitrary units (**B**). Data are presented as a mean with individual points; n = 3 technical replicates. Statistical analysis was performed using an unpaired two-tailed *t*-test. * *p*-value < 0.05.

Western blotting was performed to validate proteomic findings for ATP6V1G1 and SLC7A5, the two proteins showing the largest increases in heavy amino acid incorporation in the EC-labelled SILAC liposomes condition. ATP6V1G1 was detected as a 19 kDa band in both EC-labelled and plain SILAC liposome-treated cells, as well as in the HeLa cell lysates used as a positive control (Figure 4A). Densitometric analysis revealed a 35% increase in ATP6V1G1 signal in EC-labelled SILAC liposome-treated cells compared to plain SILAC liposomes (*p* < 0.05) (Figure 4B).

**Figure 5:**
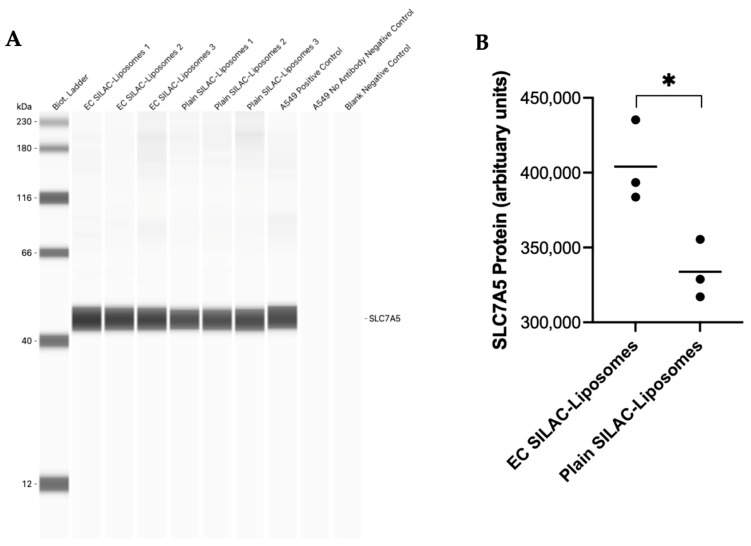
Western blot analysis and quantification of SLC7A5 protein expression. Western blot analysis of SLC7A5 in BeWo cell lysates following treatment with EC-labelled SILAC liposomes or plain SILAC liposomes, performed using Jess Simple Western (**A**). A549 cell lysate served as a positive control, while A549 cell lysate without SLC7A5 antibody incubation and a no-sample control served as negative controls. 0.4 mg/mL protein concentration and a 1:100 SLC7A5 antibody dilution were used. A biotinylated molecular weight ladder confirmed a 45 kDa SLC7A5 band. Quantification of SLC7A5, normalised to total protein, was performed using Compass for Simple Western software, with results displayed in arbitrary units (**B**). Data represent the mean with individual points; n = 3 technical replicates. Statistical analysis was performed using an unpaired two-tailed *t*-test. * *p*-value < 0.05.

Similarly, SLC7A5 was detected as a 45 kDa band in both treatment conditions and in A549 cell lysates used as a positive control (Figure 5A). Quantification showed a 19% increase in SLC7A5 signal in the EC-labelled SILAC liposome-treated cells compared to plain SILAC liposomes (*p* < 0.05) (Figure 5B). These findings independently support the proteomic evidence of increased amino acid incorporation following targeted delivery.

### 3.6. Functional Context of Proteomic Changes

**Figure 6:**
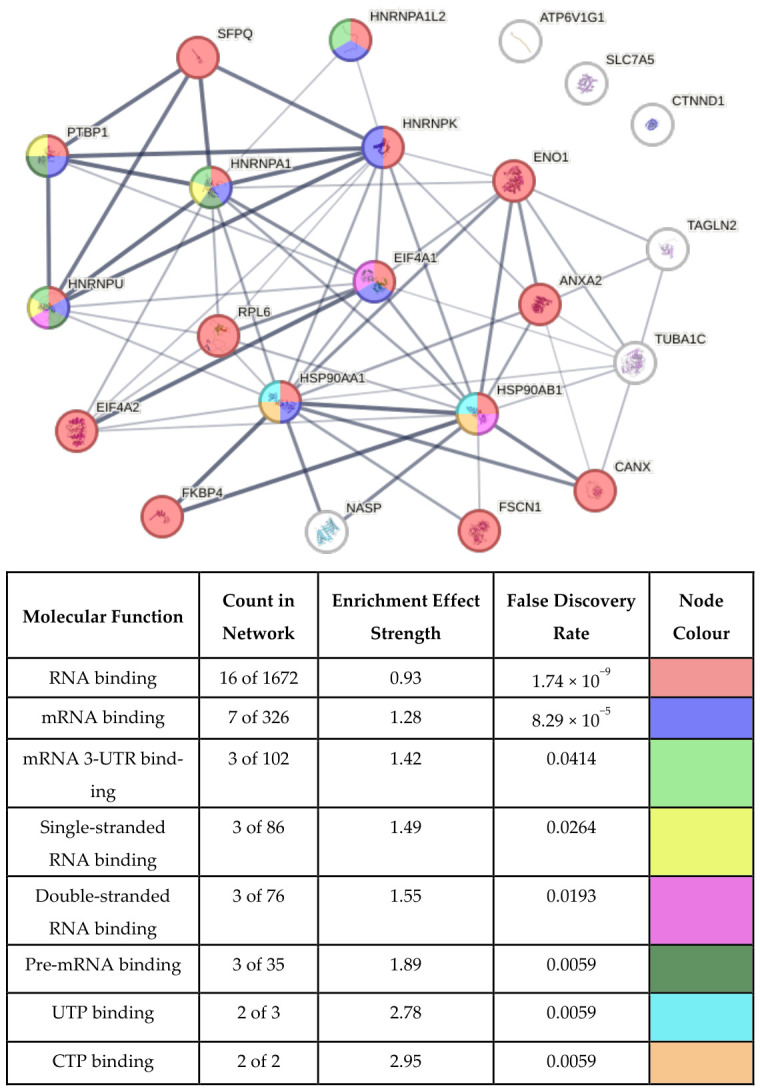
Functional enrichment of proteins in protein synthesis molecular functions. The figure illustrates proteins (nodes) in the network that are functionally enriched in protein synthesis-related molecular functions. The accompanying table presents the number of proteins in the network that are annotated with the function vs. the number of proteins in the database that are annotated with the function, the enrichment effect strength (calculated as the log10 ratio of the observed vs. expected number of proteins for a random network of the same size), the false discovery rate (shown as a Benjamini-Hochberg corrected *p*-value), and the corresponding node colours in the network. Enrichment analysis was conducted using the STRING biological database.

To provide descriptive biological context for the observed proteomic changes, functional enrichment analysis was performed on proteins showing significantly increased heavy amino acid incorporation in the EC-labelled SILAC liposome condition. Enrichment highlighted molecular functions related to protein synthesis, including RNA and mRNA binding and nucleotide binding activities (Figure 6). Proteins associated with these functions included heterogeneous nuclear ribonucleoproteins (HNRNPA1, HNRNPA1L2, HNRNPK, HNRNPU and PTBP1), heat shock proteins (HSP90AA1 and HSP90AB1), eukaryotic initiation factors (EIF4A1 and EIF4A2), a splicing factor (SFPQ), a ribosomal protein (RPL6), transcription regulators (ENO1 and ANXA2), protein chaperones (FKBP4 and CANX), and an actin-binding protein (FSCN1).

**Figure 7:**
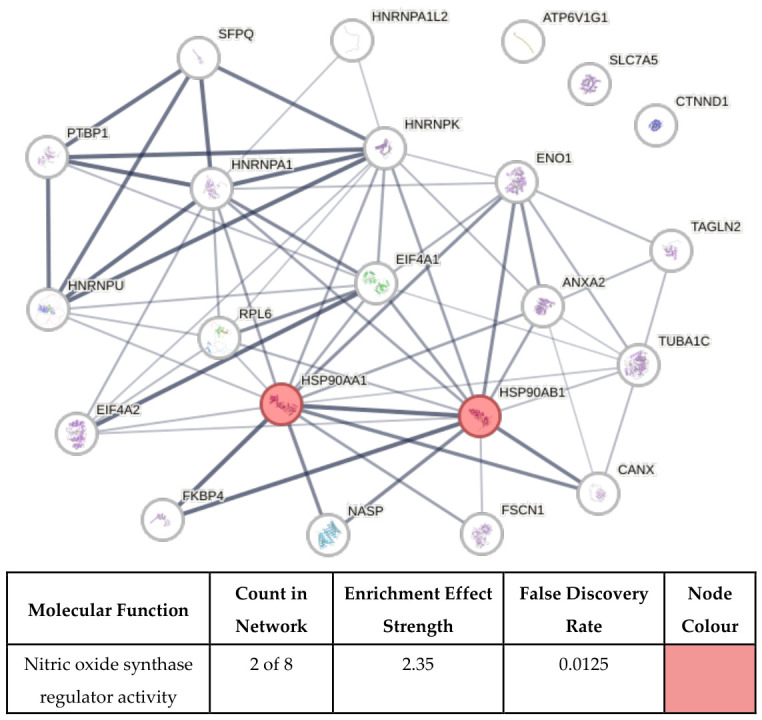
Functional enrichment of proteins in nitric oxide synthase regulator activity molecular function. The figure illustrates proteins (nodes) in the network that are functionally enriched in nitric oxide synthase regulator activity molecular function. The accompanying table presents the number of proteins in the network that are annotated with the function vs. the number of proteins in the database that are annotated with the function, the enrichment effect strength (calculated as the log10 ratio of the observed vs. expected number of proteins for a random network of the same size), the false discovery rate (shown as a Benjamini-Hochberg corrected *p*-value), and the corresponding node colours in the network. Enrichment analysis was conducted using the STRING biological database.

In addition, a limited enrichment of proteins annotated with nitric oxide synthase regulator activity was observed, specifically involving the heat shock proteins HSP90AA1 and HSP90AB1 (Figure 7). Given the inclusion of arginine among the delivered amino acids, the finding is biologically plausible. However, as nitric oxide synthase activity and nitric oxide production were not directly assessed, this finding is presented as contextual rather than indicative of pathway activation.

Additional analyses, including protein interaction network analysis, functional enrichment of protein synthesis biological processes and subcellular localisation, and co-expression analyses are provided in the Supplementary Material (Figures S5–S8).

## 4. Discussion

This study demonstrates that placenta-targeted liposomes can deliver amino acids intracellularly and result in measurable incorporation into the proteome of trophoblast-like cells. By combining EC-peptide-targeted liposomal delivery with SILAC-based quantitative proteomics, we show that L-arginine and L-lysine can be taken up via targeted intracellular delivery and incorporated into newly synthesised proteins. These findings provide proof-of-concept evidence that peptide-functionalised liposomes enable selective delivery to placental cells and induce coordinated, biologically plausible proteomic changes associated with amino acid utilisation. This framework supports further investigation of targeted nutrient delivery strategies in placental biology without reliance on endogenous amino acid transport alone.

### 4.1. Liposome Characterisation and Trophoblast Cell Targeting

DiI-labelled liposomes were characterised for morphology, size, and surface charge to assess their suitability for placental targeting. TEM revealed roughly spherical, ovoid structures with diameters of approximately 100 nm, consistent with previous reports [51,52]. However, conventional negative staining TEM has limitations in definitively confirming liposome structure, and cryo-TEM would provide improved structural characterisation in future studies.

NTA analysis showed a mean modal size of 128.2 nm ± 5.49 nm, within the target range of <150 nm, selected to balance liposome stability with efficient cellular uptake [53]. As expected for formulations prepared using this method, some size heterogeneity was observed [56]. Dynamic light scattering and polydispersity index measurements would provide complementary characterisation of particle size distribution, although these were not performed in the present study.

Surface charge is a key determinant of liposome stability and cellular uptake. Negatively charged liposomes are favoured for targeted delivery as they reduce non-specific electrostatic interactions with the cell membrane and promote receptor-mediated uptake [54,55]. In this study, plain liposomes exhibited a negative zeta potential (−22.42 mV), which became more negative following EC-labelling (−31.32 mV) and scrEC-labelling (−30.42 mV), whereas R8-labelling resulted in a positive surface charge (+22.05 mV). The observed negative zeta potential of the plain liposomes is consistent with the lipid composition, as zwitterionic phospholipids and PEGylated lipids typically exhibit a slightly negative surface potential under aqueous conditions due to interfacial charge distribution. The cationic R8 peptide drives non-specific, electrostatic internalisation across many cell types, while negatively charged EC liposomes achieve selective uptake via receptor-mediated binding [54,55].

Zeta potential measurements are influenced by ionic strength and buffer composition, as increased salt concentrations can affect the measured surface potential. In addition, dilution into low-conductivity media is commonly used to minimise ionic screening effects during measurement, although this may influence liposome stability and apparent size distribution. Furthermore, polydispersity can affect the accuracy of zeta potential measurements. However, in this study, measurements were performed under consistent conditions across all samples, allowing for reliable relative comparisons between formulations.

Consistent with the zeta potential findings, fluorescence microscopy demonstrated enhanced uptake of EC-labelled DiI liposomes in BeWo trophoblast-like cells, while scrambled and plain controls showed minimal internalisation. The uptake of R8-labelled liposomes was high, reflecting non-specific internalisation driven by surface charge. These findings align with previous in vivo reports of EC-peptide placental specificity and support the mechanistic rationale for targeted delivery to trophoblast cells [30].

SILAC liposomes were prepared using a previously established microfluidic method and lipid composition, achieving an encapsulation efficiency of 36% for L-arginine and L-lysine [47]. While sufficient for proof-of-concept, further optimisation of formulation and preparation parameters could improve encapsulation efficiency and delivery performance for future translational applications [57,58,59,60].

### 4.2. SILAC Proteomics Analysis

SILAC-based quantitative proteomics revealed differences in the incorporation of isotope-labelled amino acids between cells treated with EC-labelled and plain SILAC liposomes. Although overall levels of heavy amino acid incorporation were similar across conditions, statistically significant differences were detected at the level of individual proteins, indicating that targeted delivery influences amino acid utilisation in a selective manner. Notably, ATP6V1G1 and SLC7A5 showed modest but statistically significant increases in heavy amino acid incorporation following EC-targeted delivery. These proteins were consistently identified across complementary analytical approaches, including one-way ANOVA, volcano plot analysis, and post-hoc statistical testing, supporting the robustness of the observed differences.

ATP6V1G1, a subunit of the vacuolar ATPase, plays a central role in intracellular acidification, vesicular trafficking, and metabolic regulation, with increased expression previously associated with enhanced metabolism in proliferative contexts [61,62,63]. Its increased incorporation following EC-labelled SILAC liposome treatment is therefore consistent with an adaptive cellular response to increased intracellular amino acid availability. Similarly, SLC7A5, an L-type neutral amino acid transporter subunit involved in protein synthesis and mTOR pathway activation, exhibited increased incorporation, suggesting a coordinated cellular response to support increased amino acid utilisation and downstream anabolic signalling [64,65,66,67].

Beyond these proteins, 21 additional proteins involved in translation initiation, RNA binding, and protein folding also showed increased incorporation in the EC-labelled SILAC liposome condition. The enrichment of these functionally related proteins suggests that targeted intracellular amino acid delivery elicits a coordinated intracellular response rather than isolated protein-level effects [68,69,70]. Overall, these data demonstrate that peptide-guided liposomal delivery of amino acids can be detected at the proteomic level in trophoblast-like cells and provide a rationale for further optimisation of delivery systems and investigation of downstream functional consequences.

### 4.3. Western Blot Validation

Western blotting was used as an independent approach to support the proteomic findings for ATP6V1G1 and SLC7A5, the two proteins showing the greatest increases in heavy amino acid incorporation following EC-labelled SILAC liposome treatment. Consistent with proteomic data, both proteins showed modest but statistically significant increases in signal intensity in EC-labelled SILAC liposome-treated cells compared with plain SILAC liposome-treated cells. These findings provide orthogonal support for the SILAC-based observations and indicate that targeted intracellular amino acid delivery is associated with detectable changes at the protein level. Together, the combined proteomic and immunodetection data reinforce the conclusion that peptide-guided liposomal delivery influences intracellular amino acid utilisation in trophoblast-like cells.

### 4.4. Functional Enrichment Analysis

Functional enrichment analysis was performed to provide descriptive biological context for proteins showing increased amino acid incorporation following EC-labelled SILAC liposome treatment. Enrichment highlighted molecular functions associated with protein synthesis, including RNA processing and translation-related activities. These categories are consistent with the expected cellular utilisation of delivered amino acids and support the interpretation that targeted delivery preferentially contributes to protein synthesis-related processes under baseline conditions [71].

These observations are broadly consistent with prior work showing that amino acid supplementation, particularly with L-arginine, can stimulate protein synthesis and cellular proliferation through mTOR activation [33,34,35]. In contrast to free amino acid supplementation, the approach used here focuses on targeted intracellular delivery, highlighting the potential of peptide-guided liposomes as a platform for targeted nutrient delivery that does not rely solely on endogenous amino acid transport [4,5,6,41,72].

A limited enrichment of proteins annotated with nitric oxide synthase (NOS) regulator activity was also observed. Given that arginine serves as a substrate for NOS, this finding is biologically plausible in the context of increased intracellular arginine availability following EC-labelled liposome delivery [36]. Nitric oxide plays an important role in placental vascular regulation, and therefore alterations in NOS-associated pathways may be relevant to placental function [73,74]. However, as NOS activity and nitric oxide production were not directly assessed, this observation is presented as contextual rather than mechanistic. Direct functional studies would be required to determine whether targeted arginine delivery influences nitric oxide signalling or related vascular pathways.

### 4.5. Study Limitations and Future Directions

This study focused on L-arginine and L-lysine due to their suitability for SILAC-based proteomics [42]. While both amino acids are relevant to placental physiology, the delivery of additional essential and semi-essential amino acids was not explored and may elicit broader proteomic effects [75,76,77,78]. Dose-dependent responses were also not assessed and represent an important area for future investigation.

While this study establishes intracellular delivery and associated proteomic responses, complementary functional assays would be required to determine whether these changes translate into alterations in protein synthesis, transporter activity, or nitric oxide signalling.

All experiments were conducted in BeWo trophoblast-like cells under baseline conditions. While this model enables controlled mechanistic studies, it does not capture the complexity of the human placenta in vivo [31,32]. Accordingly, extension to additional placental models and in vivo systems will be required to evaluate functional relevance and translational potential. The previously reported placental accumulation of the EC peptide in vivo supports the feasibility of such approaches [30].

Finally, while liposomes were prepared using a defined lipid composition and an established microfluidic method, direct verification of lipid ratios following formulation (e.g., by HPLC) was not performed and represents a limitation of the current study. In addition, the stability of the liposome formulation over time was not directly assessed and represents an important area for future investigation, particularly in the context of long-term storage and translational applications.

## 5. Conclusions

In conclusion, this study demonstrates that placenta-targeted EC-labelled liposomes enable intracellular delivery of amino acids to trophoblast-like cells, with incorporation into the cellular proteome detected using SILAC-based quantitative proteomics.

Together, these findings establish a proof-of-concept platform for studying targeted nutrient delivery in placental cell biology. The observed proteomic responses are consistent with increased amino acid utilisation and protein synthesis-related processes. While functional and disease-specific outcomes were not assessed, this work provides a foundation for future studies aimed at optimizing delivery strategies and exploring downstream biological and translational relevance in placental research.

## Figures and Tables

**Table 1:** Zeta potential measurements of DiI liposomes. Zeta potential measurements for plain DiI liposomes and DiI liposomes labelled with EC, scrEC, and R8 peptides are presented. Measurements were obtained from three preparations, with mean values and standard deviation (SD) reported. Analysis was conducted using ZetaView.

DiI Liposome Preparation	Zeta Potential (mV)	SD
Plain	−22.42	1.97
EC-labelled	−31.32	2.72
scrEC-labelled	−30.42	1.49
R8-labelled	+22.05	2.10

**Table 2:** Encapsulation efficiency of SILAC amino acids in liposomes. Encapsulated and free (non-encapsulated) SILAC amino acid concentrations were measured using an L-amino acid quantitation kit, with concentrations reported in millimolar (mM). Encapsulation efficiency (%) was calculated as the proportion of amino acids encapsulated inside the liposomes relative to the total amino acids (encapsulated plus free), expressed as a percentage.

Encapsulated SILAC Amino Acid Concentration (mM)	Free (Non-Encapsulated) SILAC Amino Acid Concentration (mM)	Encapsulation Efficiency (%)
11.7	33.0	36

**Table 3:** Volcano plot analysis of EC-labelled vs. plain SILAC liposome conditions. The table presents proteins with a statistically significant log2 fold-change in heavy-to-light amino acid ratios between EC-labelled and plain SILAC liposome conditions, as identified in the volcano plot analysis. Both the log2 fold-change values (with positive values indicating fold increases and negative values indicating reciprocal fold decreases) and the corresponding linear fold-change equivalents are shown. Statistical significance is expressed as −log10 *p*-value, with raw *p*-values also provided. Proteins with an increased difference in their heavy-to-light amino acid ratio are highlighted in red, while proteins with a decreased difference are highlighted in green. Volcano plot data were generated using Perseus software.

Gene Name	Protein Name	Log2 Fold-Change Difference	Linear Fold-Change Equivalent	−Log *p*-Value	Raw *p*-Value
ATP6V1G1	V-type proton ATPase subunit G 1	1.489	2.807	3.658	0.00022
SLC7A5	Large neutral amino acid transporter small subunit 1	0.207	1.154	2.066	0.008602
SFPQ	Splicing factor, proline- and glutamine-rich	0.140	1.102	3.732	0.000185
HNRNPU	Heterogeneous nuclear ribonucleoprotein U	0.137	1.100	1.673	0.021197
PFKP	ATP-dependent 6-phosphofructokinase, platelet type	0.133	1.097	1.158	0.069617
FKBP4	Peptidyl-prolyl cis-trans isomerase FKBP4	0.130	1.094	1.550	0.028183
HSP90AB1	Heat shock protein HSP 90-beta	0.113	1.081	2.280	0.005248
ENO1	Alpha-enolase	0.112	1.081	1.953	0.011132
NASP	Nuclear autoantigenic sperm protein	0.108	1.078	2.113	0.007701
PA2G4	Proliferation-associated protein 2G4	−0.167	0.891	1.247	0.056477
NACA	Nascent polypeptide-associated complex subunit alpha	−0.336	0.792	1.137	0.072862
RPL18A	60S ribosomal protein L18a	−0.382	0.767	1.769	0.017027
BCAM	Basal cell adhesion molecule	−1.574	0.336	3.979	0.000105

**Table 4:** Post-hoc Tukey’s test results for EC-labelled SILAC liposomes vs. plain SILAC liposomes. The table presents proteins that showed a statistically significant increase in the heavy-to-light amino acid ratio in the EC-labelled SILAC liposome condition compared to the plain SILAC liposome condition, as identified through one-way ANOVA and post-hoc Tukey’s test. The table includes the gene name, protein name, and statistical significance, represented by *p*-values and q-values from the one-way ANOVA analysis. One-way ANOVA and post-hoc Tukey’s test were carried out using Perseus software.

Gene Name	Protein Name	ANOVA *p*-Value	ANOVA q-Value
ANXA2; ANXA2P2	Annexin A2; Putative annexin A2-like protein	0.0004	0.05882
ATP6V1G1	V-type proton ATPase subunit G 1	0.00409	0.04167
CANX	Calnexin	0.00307	0.04136
CTNND1	Catenin delta-1	0.00006	0.1144
EIF4A1; EIF4A2	Eukaryotic initiation factor 4A-I; Eukaryotic initiation factor 4A-II	0.00222	0.04562
ENO1	Alpha-enolase	0.00133	0.04229
FKBP4	Peptidyl-prolyl cis-trans isomerase FKBP4	0.00356	0.04112
FSCN1	Fascin	0.00006	0.143
HNRNPA1; HNRNPA1L2	Heterogeneous nuclear ribonucleoprotein A1; Heterogeneous nuclear ribonucleoprotein A1-like 2	0.0027	0.04238
HNRNPK	Heterogeneous nuclear ribonucleoprotein K	0.0006	0.05374
HNRNPU	Heterogeneous nuclear ribonucleoprotein U	0.0024	0.04328
HSP90AA1	Heat shock protein HSP 90-alpha, family class A, member 1	0.00108	0.04297
HSP90AB1	Heat shock protein HSP 90-alpha, family class B, member 1	0.00052	0.0618
NASP	Nuclear autoantigenic sperm protein	0.00092	0.04483
PTBP1	Polypyrimidine tract-binding protein 1	0.00605	0.04702
RPL6	60S ribosomal protein L6	0.00029	0.05013
SFPQ	Splicing factor, proline- and glutamine-rich	0.0009	0.04557
SLC7A5	Large neutral amino acid transporter small subunit 1	0.00366	0.04149
TAGLN2	Transgelin-2	0.00121	0.0455
TUBA1C	Tubulin alpha-1C chain	0.00045	0.06126

## Data Availability

The original contributions presented in this study are included in the article/Supplementary Material. Further inquiries can be directed to the corresponding author.

## Supplementary Materials

The following supporting information can be downloaded at: https://www.mdpi.com/article/10.3390/biom16050628/s1, Figure S1. Histograms of EC-labelled SILAC liposomes, plain SILAC liposomes, and SILAC medium control triplicates. Heavy-to-light amino acid ratio data for 711 proteins across triplicate samples in each experimental condition were visualised as histograms to evaluate whether the data followed a normal distribution after log2 transformation. X-axis represents the log2 heavy-to-light ratios, Y-axis indicates their frequency. Histograms were generated using Perseus software; Figure S2. Principal component analysis (PCA) of EC-labelled SILAC liposomes, plain SILAC liposomes, and SILAC medium control triplicates. PCA was performed on heavy-to-light ratio data for 711 proteins across triplicate samples in each experimental condition to assess data similarity between replicates and differences between treatment groups. X-axis represents Principal Component 1, which accounts for the greatest variance in the dataset. Y-axis represents Principal Component 2, which accounts for the second greatest variance in the dataset. EC-labelled SILAC liposomes triplicates are highlighted in red, plain SILAC liposomes in blue, and SILAC medium controls in green. PCA plot was generated using Perseus software; Figure S3. Volcano plot analysis of EC-labelled vs. plain SILAC liposomes conditions. The volcano plot displays log2 fold-change differences in heavy-to-light amino acid ratios between EC-labelled and plain SILAC liposomes conditions. Analysis includes all proteins identified as statistically significant in a one-way ANOVA test. X-axis represents the log2 fold-change in heavy-to-light amino acid ratio, Y-axis represents statistical significance as −log10 *p*-values. Analysis parameters: S_0_ = 0.1, false discovery rate = 0.05. Significantly differentially incorporated proteins (red) are labelled with gene symbols. The volcano plot was generated using Perseus software; Figure S4. Heat map of heavy-to-light amino acid ratios across conditions. The heat map shows proteins with significant differences in log2 heavy-to-light ratios between EC-labelled SILAC liposomes, plain SILAC liposomes, and SILAC medium control-treated cells, based on one-way ANOVA results. Z-score transformation was applied for normalisation, and hierarchical clustering identified three distinct protein groups. Red indicates an increase in heavy-to-light amino acid ratio, while green indicates a decrease in the heavy-to-light amino acid ratio. The heat map was generated using Perseus software; Table S1. Number of unique peptides and representative peptide sequences for proteins with increased heavy amino acid incorporation in the EC-labelled SILAC liposomes condition. The table lists proteins with a statistically significant increase in heavy-to-light amino acid ratio in the EC-labelled SILAC liposomes condition compared to the plain SILAC liposomes condition. For each protein, the number of unique peptides and representative peptide sequences identified by mass spectrometry are provided; Figure S5. Protein interaction network of significantly enriched proteins in the EC-labelled SILAC liposomes condition. The network illustrates proteins with a statistically significant increase in heavy-to-light amino acid ratio in the EC-labelled SILAC liposomes condition compared to the plain SILAC liposomes condition. Proteins are represented as nodes, while edges indicate interactions between them. Edge thickness corresponds to interaction confidence, ranging from 0.15 (low confidence, thinner lines), to 0.9 (high confidence, thicker lines). Coloured nodes represent the queried proteins; the specific hue of a coloured node has no biological or quantitative meaning and is used solely for visual distinction. The figure also displays the number of nodes, number of edges, expected number of edges for a network of this size, protein-protein interaction (PPI) enrichment *p*-value, average node degree, and average local clustering coefficient. The network was generated using the STRING (Search Tool for the Retrieval of Interacting Genes/proteins) biological database; Figure S6. Functional enrichment of proteins in protein synthesis biological processes. The figure illustrates proteins (nodes) in the network that are functionally enriched in protein synthesis-related biological processes. The accompanying table presents the number of proteins in the network that are annotated with the process vs. the number of proteins in the database that are annotated with the process, the enrichment effect strength (calculated as the log10 ratio of observed vs. expected number of proteins for a random network of the same size), the false discovery rate (shown as a Benjamini-Hochberg corrected *p*-value), and the corresponding node colours in the network. Enrichment analysis was conducted using the STRING biological database; Figure S7. Functional enrichment of proteins in protein synthesis subcellular localisations. Figure illustrates proteins (nodes) in the network that are functionally enriched in protein synthesis-related subcellular localisations. The accompanying table presents the number of proteins in the network that are annotated with the localisation vs. the number of proteins in the database that are annotated with the localisation, the enrichment effect strength (calculated as the log10 ratio of observed vs. expected number of proteins for a random network of the same size), the false discovery rate (shown as a Benjamini-Hochberg corrected *p*-value), and the corresponding node colours in the network. Enrichment analysis was conducted using the STRING biological database; Figure S8. Co-expression analysis of significantly enriched proteins in the EC-labelled SILAC liposomes condition. The figure illustrates protein co-expression patterns for proteins that showed a statistically significant increase in heavy SILAC amino acid incorporation in the EC-labelled SILAC liposomes condition compared to plain SILAC liposomes condition. Darker red colours indicate higher co-expression scores between protein pairs. The accompanying table lists protein pairs with co-expression scores above 0.5, along with their shared biological function. Co-expression analysis was performed using the STRING biological database and was based on RNA expression patterns.

## Abbreviations

The following abbreviations are used in this manuscript: AGC: Automatic gain control; ANXA2/P2: Annexin A2/A2-like; ATP6V1G1: V-type proton ATPase subunit G1; BCA: Bicinchoninic acid; BCAM: Basal cell adhesion molecule; CANX: Calnexin; CTNND1: Catenin delta-1; DiI: 1′-Dioctadecyl-3,3,3′,3′-tetramethylindocarbocyanine perchlorate; DMEM: Dulbecco’s Modified Eagle’s Medium; EC: EDVKDINFDTKEKFLAGCLIVSFHEGKC peptide (also known as plCSA-bp); EDTA: Ethylenediaminetetraacetic acid; EIF4A1/2: Eukaryotic initiation factor 4A-I/II; ENO1: Alpha-enolase; FBS: Foetal bovine serum; FGR: Foetal growth restriction; FKBP4: Peptidyl-prolyl cis-trans isomerase FKBP4; fPBS: Filtered phosphate buffered saline; FSCN1: Fascin; HNRNPA1/L2: Heterogeneous nuclear ribonucleoprotein A1/A1-like 2; HNRNPK: Heterogeneous nuclear ribonucleoprotein K; HNRNPU: Heterogeneous nuclear ribonucleoprotein U; HSP90AA1/B1: Heat shock protein HSP 90-alpha A1/B1; HyD: Hybrid detector; K8: L-lysine-2HCl 4,4,5,5-D4 (heavy lysine isotope); LC-MS/MS: Liquid chromatography-tandem mass spectrometry; mM: Millimolar; NA: Numerical aperture; NACA: Nascent polypeptide-associated complex subunit alpha; NASP: Nuclear autoantigenic sperm protein; NOS: Nitric oxide synthase; NTA: Nanoparticle tracking analysis; PA2G4: Proliferation-associated protein 2G4; PBS: Phosphate buffered saline; PCA: Principal component analysis; PFKP: ATP-dependent 6-phosphofructokinase, platelet type; plCSA-bp: Placental chondroitin sulphate A-binding peptide (synonym for EC); PMT: Photomultiplier tube detector; PPI: Protein-protein interaction; PTBP1: Polypyrimidine tract-binding protein 1; R8: Octaarginine peptide (RRRRRRRR); R10: L-arginine-HCl 13C6 (heavy arginine isotope); RIPA: Radioimmunoprecipitation assay (lysis buffer); RPL6/18A: 60S ribosomal protein L6/L18a; scrEC: EVDNDKKLGLVFEKDKIFTEFACISHCG peptide; SD: Standard deviation; SFPQ: Splicing factor, proline- and glutamine-rich; SILAC: Stable isotope labelling by amino acids in cell culture; SLC7A5: Large neutral amino acids transporter small subunit 1; STRING: Search tool for the retrieval of interacting genes/proteins; TAGLN2: Transgelin-2; TEM: Transmission electron microscopy; TUBA1C: Tubulin alpha-1C chain.
