# Supplementary material for: Targeted Intracellular Delivery of Amino Acids to Trophoblast Cells Reveals Proteomic Signatures of Cellular Utilisation
Source: Biomolecules. 2026 Apr 23;16(5):628. doi: 10.3390/biom16050628 (PMC13205100; doi:10.3390/biom16050628)
Supplement: Supplementary file 1 [file biomolecules-16-00628-s001.zip › Figure S1.pdf]

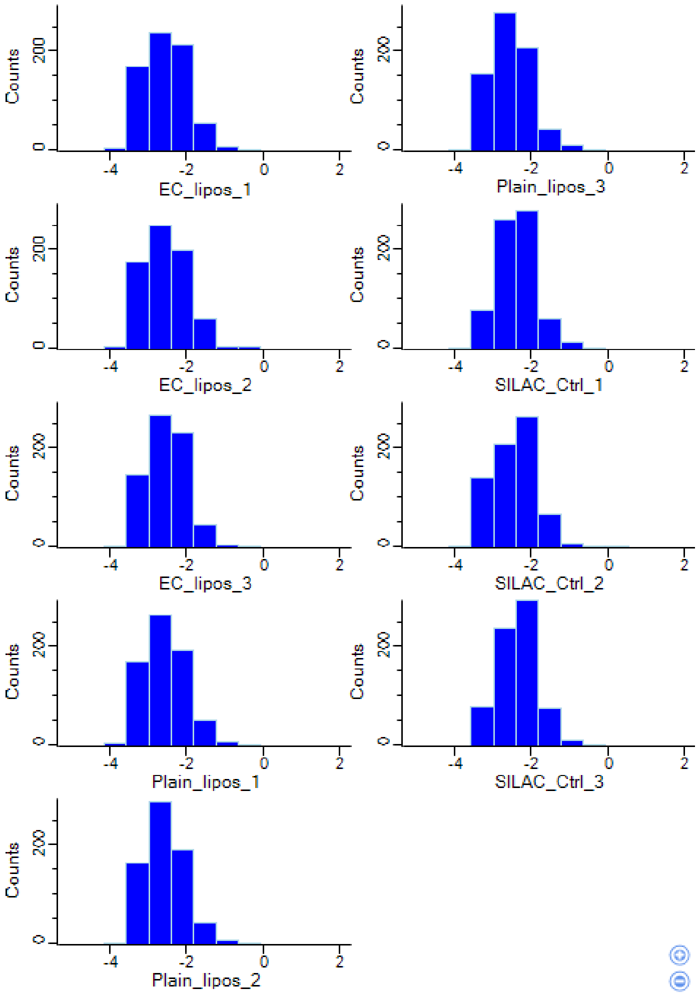

Figure S1. Histograms of EC-labelled SILAC liposomes, plain SILAC liposomes, and SILAC medium control triplicates. Heavy-to-light amino acid ratio data for 711 proteins across triplicate samples in each experimental condition were visualised as histograms to evaluate whether the data followed a normal distribution after log2 transformation. X-axis represents the log2 heavy-to-light ratios, Y-axis indicates their frequency. Histograms were generated using Perseus software.
