# Supplementary material for: Targeted Intracellular Delivery of Amino Acids to Trophoblast Cells Reveals Proteomic Signatures of Cellular Utilisation
Source: Biomolecules. 2026 Apr 23;16(5):628. doi: 10.3390/biom16050628 (PMC13205100; doi:10.3390/biom16050628)
Supplement: Supplementary file 1 [file biomolecules-16-00628-s001.zip › Figure S2.pdf]

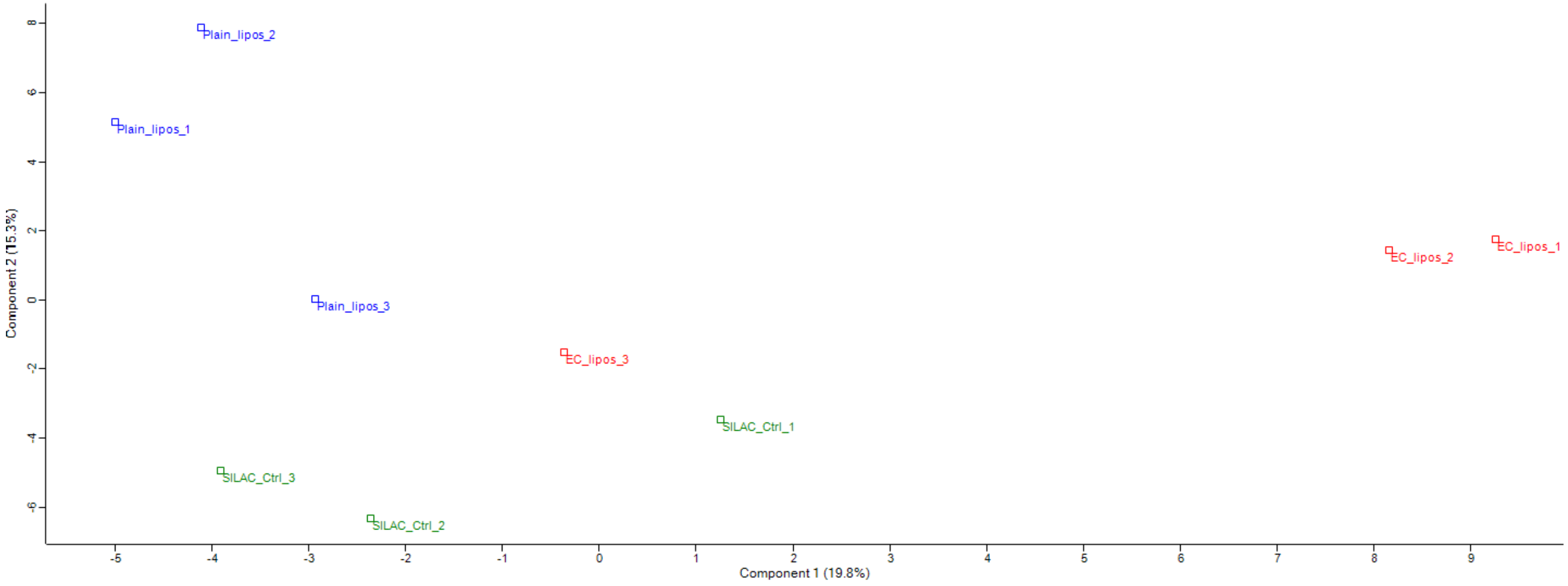

Figure S2. Principal component analysis (PCA) of EC-labelled SILAC liposomes, plain SILAC liposomes, and SILAC medium control triplicates. PCA was performed on heavy-to-light ratio data for 711 proteins across triplicate samples in each experimental condition to assess data similarity between replicates and differences between treatment groups. X-axis represents Principal Component 1, which accounts for the greatest variance in the dataset. Y-axis represents Principal Component 2, which accounts for the second greatest variance in the dataset. EC-labelled SILAC liposomes triplicates are highlighted in red, plain SILAC liposomes in blue, and SILAC medium controls in green. The PCA plot was generated using Perseus software.
