# Supplementary material for: Targeted Intracellular Delivery of Amino Acids to Trophoblast Cells Reveals Proteomic Signatures of Cellular Utilisation
Source: Biomolecules. 2026 Apr 23;16(5):628. doi: 10.3390/biom16050628 (PMC13205100; doi:10.3390/biom16050628)
Supplement: Supplementary file 1 [file biomolecules-16-00628-s001.zip › Figure S3.pdf]

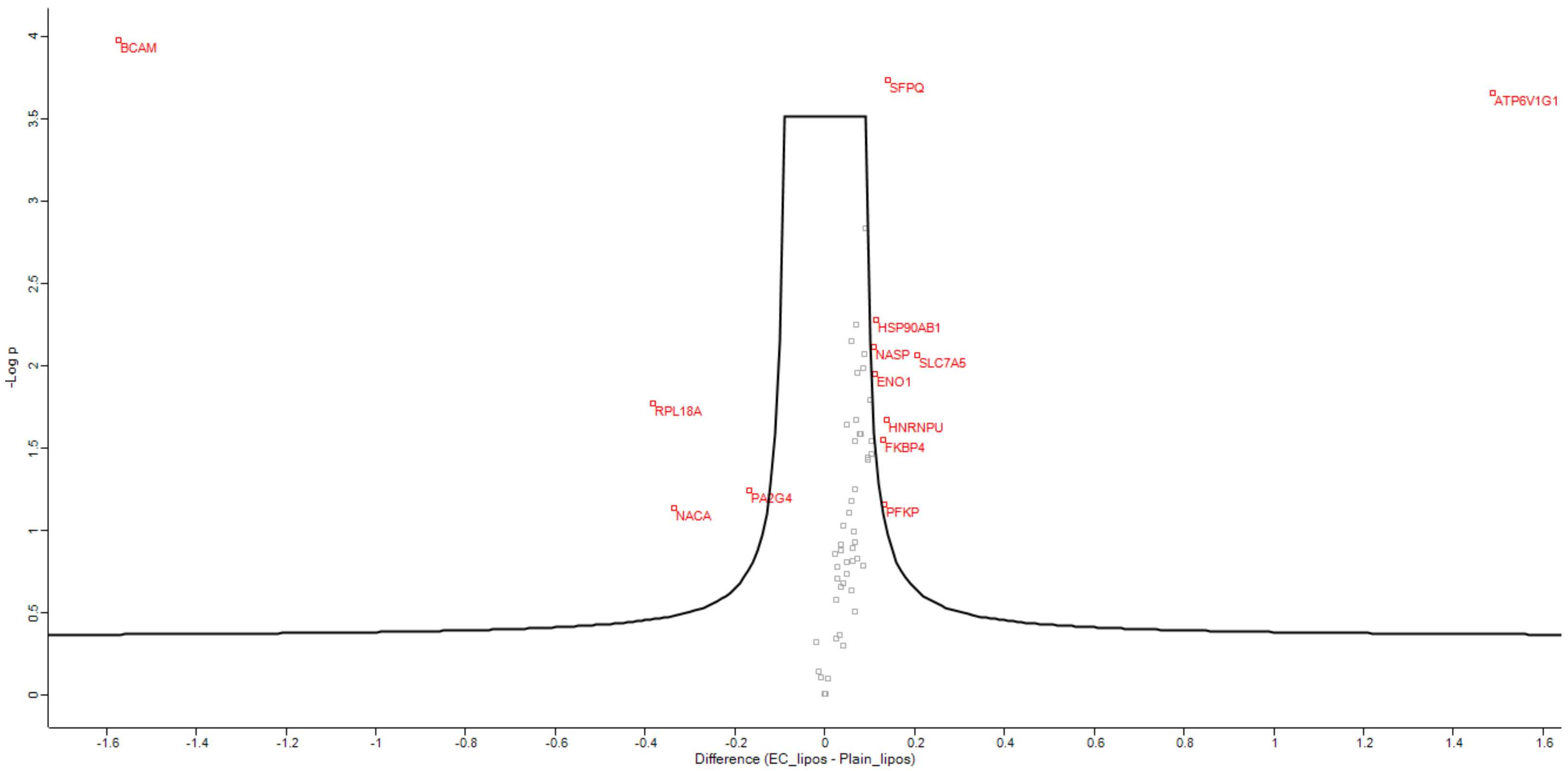

Figure S3. Volcano plot analysis of EC-labelled vs. plain SILAC liposomes conditions. Volcano plot displays fold-change differences in log2 heavy-to-light amino acid ratios between EC-labelled and plain SILAC liposomes conditions. Analysis includes all proteins identified as statistically significant in a one-way ANOVA test. X-axis represents the log2 fold-change in heavy-to-light amino acid ratio, Y-axis represents statistical significance as -log *p*-values. Analysis parameters: $S_0 = 0.1$, false discovery rate = 0.05. Significantly differentially incorporated proteins (red) are labelled with gene symbols. The volcano plot was generated using Perseus software.
