# Supplementary material for: Targeted Intracellular Delivery of Amino Acids to Trophoblast Cells Reveals Proteomic Signatures of Cellular Utilisation
Source: Biomolecules. 2026 Apr 23;16(5):628. doi: 10.3390/biom16050628 (PMC13205100; doi:10.3390/biom16050628)
Supplement: Supplementary file 1 [file biomolecules-16-00628-s001.zip › Figure S4.pdf]

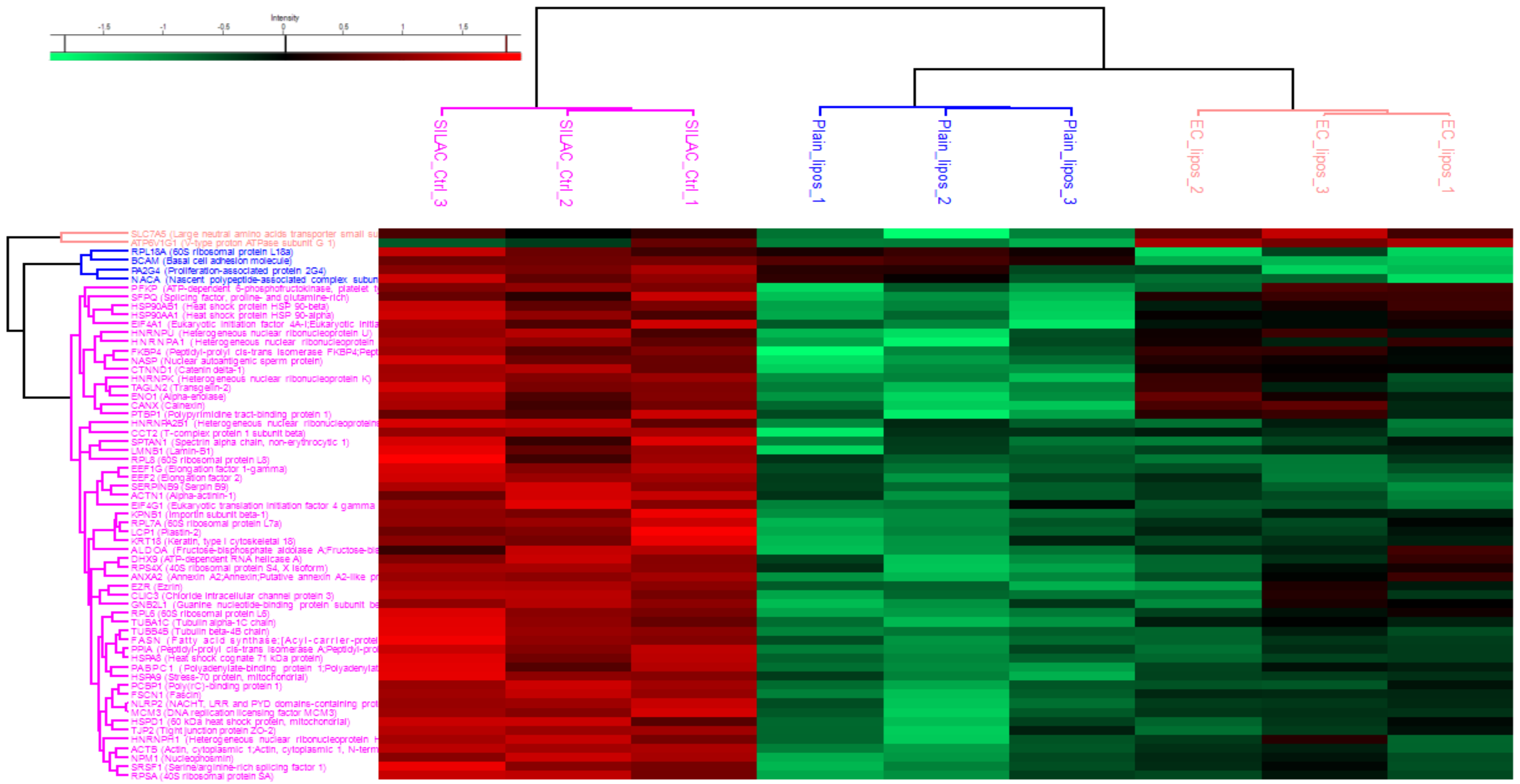

Figure S4. Heat map of heavy-to-light amino acid ratios across conditions. The heat map shows proteins with significant differences in log2 heavy-to-light ratios between EC-labelled SILAC liposomes, plain SILAC liposomes, and SILAC medium control-treated cells, based on one-way ANOVA results. Z-score transformation was applied for normalisation, and hierarchical clustering identified three distinct protein groups. Red indicates an increase in heavy-to-light amino acid ratio, while green indicates a decrease in the heavy-to-light amino acid ratio. The heat map was generated using Perseus software.
