# Supplementary material for: Targeted Intracellular Delivery of Amino Acids to Trophoblast Cells Reveals Proteomic Signatures of Cellular Utilisation
Source: Biomolecules. 2026 Apr 23;16(5):628. doi: 10.3390/biom16050628 (PMC13205100; doi:10.3390/biom16050628)
Supplement: Supplementary file 1 [file biomolecules-16-00628-s001.zip › Figure S5.pdf]

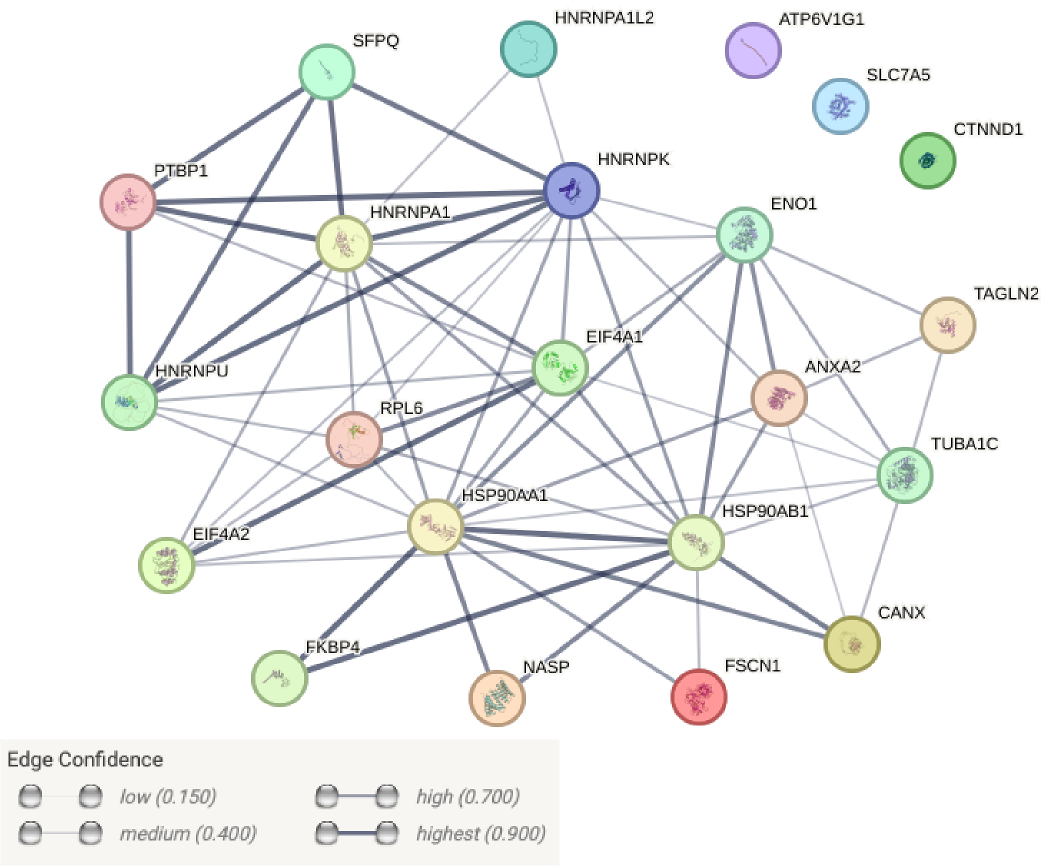

Number of nodes: 22
Number of edges: 63
Average node degree: 5.73

Expected number of edges: 17
PPI enrichment p-value: $<1 \times 10^{-16}$
Average local clustering coefficient: 0.685

Figure S5. Protein interaction network of significantly enriched proteins in the EC-labelled SILAC liposomes condition. The network illustrates proteins with a statistically significant increase in heavy-to-light amino acid ratio in the EC-labelled SILAC liposomes condition compared to the plain SILAC liposomes condition. Proteins are represented as nodes, while edges indicate interactions between them. Edge thickness corresponds to interaction confidence, ranging from 0.15 (low confidence, thinner lines), to 0.9 (high confidence, thicker lines). Coloured nodes represent the queried proteins; the specific hue of a coloured node has no biological or quantitative meaning and is used solely for visual distinction. The figure also displays the number of nodes, number of edges, expected number of edges for a network of this size, protein-protein interaction (PPI) enrichment *p*-value, average node degree, and average local clustering coefficient. The network was generated using the STRING (Search Tool for the Retrieval of Interacting Genes/proteins) biological database.
