# Supplementary material for: Targeted Intracellular Delivery of Amino Acids to Trophoblast Cells Reveals Proteomic Signatures of Cellular Utilisation
Source: Biomolecules. 2026 Apr 23;16(5):628. doi: 10.3390/biom16050628 (PMC13205100; doi:10.3390/biom16050628)
Supplement: Supplementary file 1 [file biomolecules-16-00628-s001.zip › Figure S6.pdf]

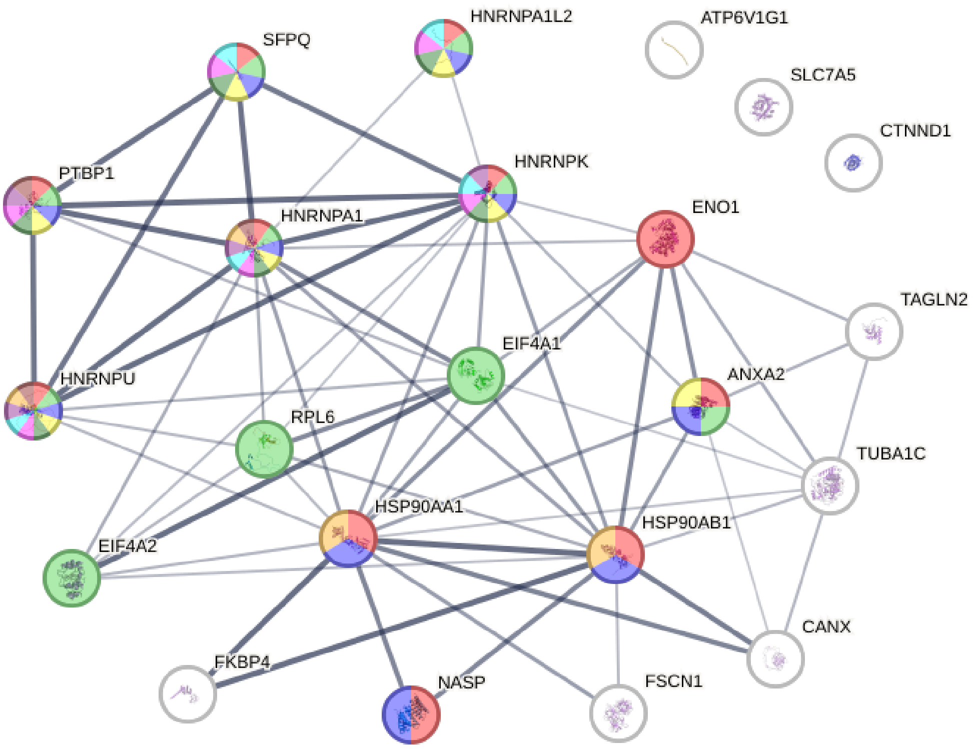

Biological Process	Count in Network	Enrichment Effect Strength	False Discovery Rate	Node Colour
Nucleobase-containing compound metabolic processes	11 of 2722	0.56	0.0443	
Nucleic acid metabolism	10 of 2203	0.61	0.0443	
Gene expression	10 of 2101	0.63	0.0388	
mRNA metabolic processes	7 of 611	1.01	0.033	
mRNA processing	6 of 455	1.07	0.033	
RNA splicing	6 of 370	1.16	0.033	
mRNA splicing, via spliceosome	5 of 245	1.26	0.033	
Regulation of DNA biosynthetic process	4 of 124	1.46	0.033	
Regulation of mRNA splicing, via spliceosome	4 of 110	1.51	0.033	
Regulation of alternative mRNA splicing, via spliceosome	3 of 61	1.64	0.0443	

Figure S6. Functional enrichment of proteins in protein synthesis biological processes. The figure illustrates proteins (nodes) in the network that are functionally enriched in protein synthesis-related biological processes. The accompanying table presents the number of proteins in the network that are annotated with the process vs. the number of proteins in the database that are annotated with the process, the enrichment effect strength (calculated as the log10 ratio of observed vs. expected number of proteins for a random network of the same size), the false discovery rate (shown as a Benjamini-Hochberg corrected *p*-value), and the corresponding node colours in the network. Enrichment analysis was conducted using the STRING biological database.
