# Supplementary material for: Targeted Intracellular Delivery of Amino Acids to Trophoblast Cells Reveals Proteomic Signatures of Cellular Utilisation
Source: Biomolecules. 2026 Apr 23;16(5):628. doi: 10.3390/biom16050628 (PMC13205100; doi:10.3390/biom16050628)
Supplement: Supplementary file 1 [file biomolecules-16-00628-s001.zip › Figure S7.pdf]

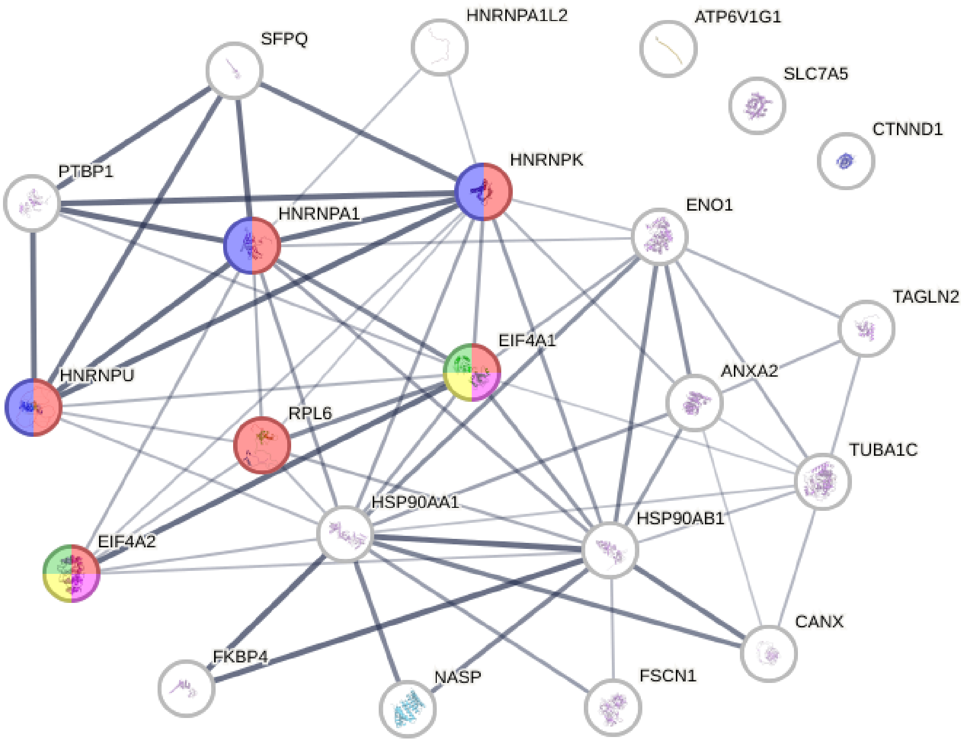

**Subcellular Localisation**	**Count in Network**	**Enrichment Effect Strength**	**False Discovery Rate**	**Node Colour**
Ribonucleoprotein complex	6 of 564	0.98	0.0055	
Catalytic step 2 spliceosome	3 of 83	1.51	0.0167	
Eukaryotic translation initiation factor 3 complex	2 of 19	1.97	0.027	
Eukaryotic translation initiation factor 4F complex	2 of 12	2.17	0.0164	
Translation initiation complex	2 of 6	2.47	0.0059	

Figure S7. Functional enrichment of proteins in protein synthesis subcellular localisations. Figure illustrates proteins (nodes) in the network that are functionally enriched in protein synthesis-related subcellular localisations. The accompanying table presents the number of proteins in the network that are annotated with the localisation vs. the number of proteins in the database that are annotated with the localisation, the enrichment effect strength (calculated as the log10 ratio of observed vs. expected number of proteins for a random network of the same size), the false discovery rate (shown as a Benjamini-Hochberg corrected *p*-value), and the corresponding node colours in the network. Enrichment analysis was conducted using the STRING biological database.
