# Supplementary material for: Targeted Intracellular Delivery of Amino Acids to Trophoblast Cells Reveals Proteomic Signatures of Cellular Utilisation
Source: Biomolecules. 2026 Apr 23;16(5):628. doi: 10.3390/biom16050628 (PMC13205100; doi:10.3390/biom16050628)
Supplement: Supplementary file 1 [file biomolecules-16-00628-s001.zip › Figure S8.pdf]

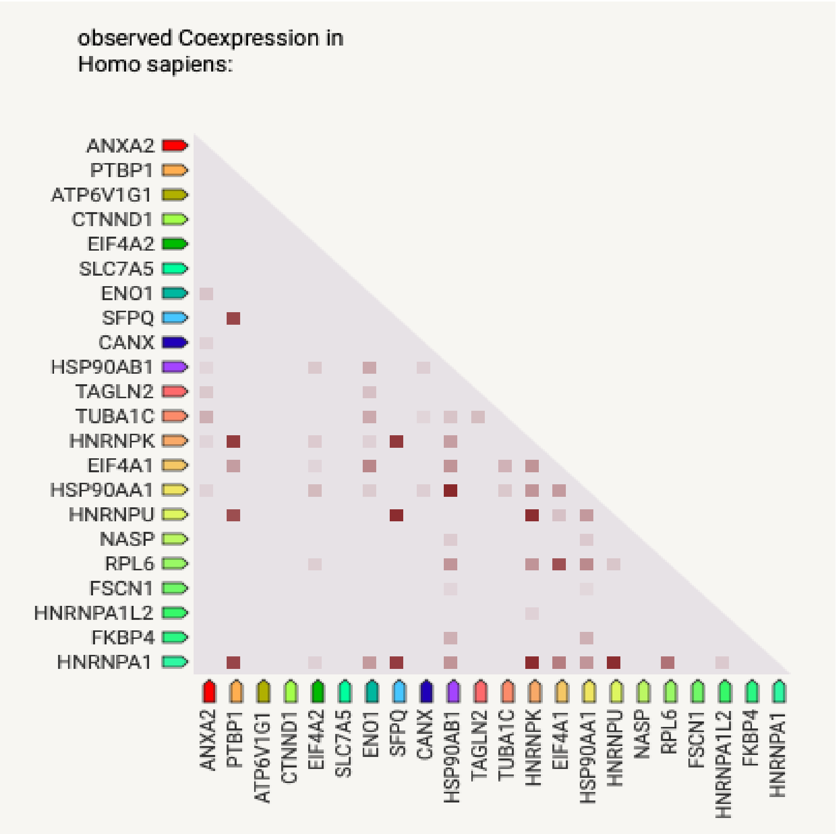


| Co-Expressed Proteins | Co-Expression Score | Function |
|---|---|---|
| HNRNPU & SFPQ | 0.783 | RNA & DNA-binding proteins (involved in transcription & translation) |
| HSP90AA1 & HSP90AB1 | 0.783 | Heat shock proteins (involved in protein homeostasis) |
| HNRNPK & HNRNPU | 0.778 | RNA- & DNA-binding proteins (involved in transcription & translation) |
| HNRNPK & HNRNPA1 | 0.778 | Pre-mRNA-binding proteins (involved in translation) |
| HNRNPA1 & HNRNPU | 0.777 | RNA- & DNA-binding proteins (involved in transcription & translation) |
| RPL6 & EIF4A1 | 0.728 | Ribosomal protein & translation initiation factor (involved in translation) |
| HNRNPK & SFPQ | 0.727 | RNA- & DNA-binding proteins (involved in transcription & translation) |
| HNRNPA1 & SFPQ | 0.708 | RNA- & DNA-binding proteins (involved in transcription & translation) |
| HNRNPK & PTBP1 | 0.707 | Pre-mRNA-binding proteins (involved in translation) |
| HNRNPA1 & PTBP1 | 0.665 | RNA-binding proteins (involved in translation) |
| PTBP1 & SFPQ | 0.663 | RNA- & DNA-binding proteins (involved in transcription & translation) |
| HNRNPU & PTBP1 | 0.631 | RNA- & DNA-binding proteins (involved in transcription & translation) |

Figure S8. Co-expression analysis of significantly enriched proteins in the EC-labelled SILAC liposomes condition. The figure illustrates protein co-expression patterns for proteins that showed a statistically significant increase in heavy SILAC amino acid incorporation in the EC-labelled SILAC liposomes condition compared to plain SILAC liposomes condition. Darker red colours indicate higher co-expression scores between protein pairs. The accompanying table lists protein pairs with co-expression scores above 0.5, along with their shared biological function. Co-expression analysis was performed using the STRING biological database and was based on RNA expression patterns.
