# Supplementary material for: Targeted Intracellular Delivery of Amino Acids to Trophoblast Cells Reveals Proteomic Signatures of Cellular Utilisation
Source: Biomolecules. 2026 Apr 23;16(5):628. doi: 10.3390/biom16050628 (PMC13205100; doi:10.3390/biom16050628)
Supplement: Supplementary file 1 [file biomolecules-16-00628-s001.zip › Table S1.pdf]

Table S1. Number of unique peptides and representative peptide sequences for proteins with increased heavy amino acid incorporation in the EC-labelled SILAC liposomes condition. The table lists proteins with a statistically significant increase in heavy-to-light amino acid ratio in the EC-labelled SILAC liposomes condition compared to the plain SILAC liposomes condition. For each protein, the number of unique peptides and representative peptide sequences identified by mass spectrometry are provided.

Gene Name	Protein Name	Unique Peptides	Representative Peptide Sequences
ANXA2; ANXA2P2	Annexin A2; Putative annexin A2-like protein	26	AEDGSVIDYELIDQDAR; AYTNFDAER; DALNIETAIK; DIISDTSGDFR; DLYDAGVK; GDLENAFLNLVQCIQNK
ATP6V1G1	V-type proton ATPase subunit G 1	3	DEVLDNLLAFVCDIRPEIHENYR; EEAQAEIEQYR; MTILQTYFR
CANX	Calnexin	27	AEEDEILNR; APVPTGEVYFADSFDR; CESAPGCGVWQR; DDTDDEIAK; DKGDEEEEGEEKLEEK; EIEDPEDR
CTNND1	Catenin delta-1	24	ALSAIADLLTNEHER; DMDLTEVITGTLWNLSSHDSIK; EEIQMSNMGSNTK; FHPEPYGLEDDQR; GIPVLVGLLDHPK; GYELLFQPEVVR
EIF4A1; EIF4A2	Eukaryotic initiation factor 4A-I; Eukaryotic initiation factor 4A-II	18	AILPCIK; ATQALVLAPTR; DFTVSAMHGDMDQK; DQIYDIFQK; ELAQQIQK; GIDVQQVSLVINYDLPTNR
ENO1	Alpha-enolase	23	AAVPSGASTGIYEALELR; DATNVGDEGGFAPNILENK; DYPVVSIEDPFDQDDWGAWQK; EGLELLK; FGANAILGVSLAVCK; FTASAGIQVVGDDLTVTNPK
FKBP4	Peptidyl-prolyl cis-tran isomerase FKBP4	23	AEASSGDHPTDTEMKEEQK; AKESWEMNSEEK; ALELDSNNEK; ATESGAQSAPLPMEGVDISPK; AWDIAIATMK; EGTGTEMPMIGDR
FSCN1	Fascin	28	ASAETVDPASLWEY; DELFALEQSCAQVVLQAANER; DVPWGVDSLITLAFQDQR; EVPGPDCR; FLIVAHDDGR; GEHGFIGCR
HNRNPA1; HNRNPA1L2	Heterogeneous nuclear ribonucleoprotein A1; Heterogeneous nuclear ribonucleoprotein A1-like 2	11	DYFEQYGK; EDSQRPGAHLTVK; EDTEEHHLR; GFAFVTFDDHDSVDK; IEVIEIMTDR; KLFIGGLSFETTDESLR
HNRNPK	Heterogeneous nuclear ribonucleoprotein K	4	AQPYDPNFYDETYDYGGFTMMFDDR; DLAGSIIGK; GGDLMAYDR; IDEPLEGSEDR
HNRNPU	Heterogeneous nuclear ribonucleoprotein U	34	AEGGGGGGRPGAPAAGDGK; AVVVCPK; DCEVVMMIGLPGAGK; DIDIHEVR; DLPEHAVLK; EKPYFPIPEEYTFIQNVPLEDR
HSP90AA1	Heat shock protein HSP 90-alpha, family class A, member 1	26	ADLINNLGTIAK; ALLFVPR; APFDLFENR; CLELFTELAEDKENYK; DNSTMGYMAAK; DQVANSAFVER
HSP90AB1	Heat shock protein HSP 90-alpha, family class B, member 1	23	ADLINNLGTIAK; AKFENLCK; ALLFIPR; APFDLFENK; CLELFSELAEDKENYK; DNSTMGYMMAK

NASP	Nuclear autoantigenic sperm protein	27	AMESTATAAVAAELVSADK; ATLVESSTSGFTPGGGGSSVSMIASR; DGAVNGPSVVGDQTPIEPQTSIER; EAQLYAAQAHLK; EDMDISK; EGEETEGSEEDDKENDKTEEMPNDSVLENK
PTBP1	Polypyrimidine tract-binding protein 1	13	AQAALQAVNSVQSGNLALAASAAAVDAGMAMAGQSPVLR; DYGNSPLHR; GQPIYIQFSNHK; HQNVQLPR; IAIPGLAGAGNSVLLVSNLNPER; IIVENLFYPVTLDVLHQIFSK
RPL6	60S ribosomal protein L6	13	AIPQLQGYLR; ASITPGTILIILTGR; AVDSQILPK; EKYEITEQR; FVIATSTK; HLTDAYFK
SFPQ	Splicing factor, proline- and glutamine-rich	25	AELDDTPMR; ANLSLLR; AVVIVDDR; CSEGVFLLTTTPR; DKLESEMEDAYHEHQANLLR; EEEMMIR
SLC7A5	Large neutral amino acids transporter small subunit 1	5	ALAAPAAEEK; ALAAPAAEEKEEAR; GDVSNLDPNFSFEGTK; LFFVGSR; SADGSAPAGEGEGVTLQR
TAGLN2	Transgelin-2	13	DDGLFSGDPNWFPK; DGTVLCELINALYPEGQAPVK; ENFQNWLK; GASQAGMTGYGMPR; IQASTMAFK; NFSDNQLQEGK
TUBA1C	Tubulin alpha-1C chain	4	AFVHWYVGEGMEEGEFSEAR; AYHEQLTVAEITNACFEPANQMVK; DYEEVGADSADGEDEGEEY; EDMAALEK
